# *PpTCP18* is upregulated by lncRNA5 and controls branch number in peach (*Prunus persica*) through positive feedback regulation of strigolactone biosynthesis

**DOI:** 10.1093/hr/uhac224

**Published:** 2022-10-07

**Authors:** Xiaobei Wang, Qiuping Wang, Lixia Yan, Yuhang Hao, Xiaodong Lian, Haipeng Zhang, Xianbo Zheng, Jun Cheng, Wei Wang, Langlang Zhang, Xia Ye, Jidong Li, Bin Tan, Jiancan Feng

**Affiliations:** College of Horticulture, Henan Agricultural University, 95 Wenhua Road, 450002, Zhengzhou, China; College of Horticulture, Henan Agricultural University, 95 Wenhua Road, 450002, Zhengzhou, China; College of Horticulture, Henan Agricultural University, 95 Wenhua Road, 450002, Zhengzhou, China; College of Horticulture, Henan Agricultural University, 95 Wenhua Road, 450002, Zhengzhou, China; College of Horticulture, Henan Agricultural University, 95 Wenhua Road, 450002, Zhengzhou, China; College of Horticulture, Henan Agricultural University, 95 Wenhua Road, 450002, Zhengzhou, China; College of Horticulture, Henan Agricultural University, 95 Wenhua Road, 450002, Zhengzhou, China; College of Horticulture, Henan Agricultural University, 95 Wenhua Road, 450002, Zhengzhou, China; College of Horticulture, Henan Agricultural University, 95 Wenhua Road, 450002, Zhengzhou, China; College of Horticulture, Henan Agricultural University, 95 Wenhua Road, 450002, Zhengzhou, China; College of Horticulture, Henan Agricultural University, 95 Wenhua Road, 450002, Zhengzhou, China; College of Forestry, Henan Agricultural University, 95 Wenhua Road, 450002, Zhengzhou, China; College of Horticulture, Henan Agricultural University, 95 Wenhua Road, 450002, Zhengzhou, China; College of Horticulture, Henan Agricultural University, 95 Wenhua Road, 450002, Zhengzhou, China

## Abstract

Branch number is an important agronomic trait in peach (*Prunus persica*) trees because plant architecture affects fruit yield and quality. Although breeders can select varieties with different tree architecture, the biological mechanisms underlying architecture remain largely unclear. In this study, a pillar peach (‘Zhaoshouhong’) and a standard peach (‘Okubo’) were compared. ‘Zhaoshouhong’ was found to have significantly fewer secondary branches than ‘Okubo’. Treatment with the synthetic strigolactone (SL) GR24 decreased branch number. Transcriptome analysis indicated that *PpTCP18* (a homologous gene of *Arabidopsis thaliana BRC1*) expression was negatively correlated with strigolactone synthesis gene expression, indicating that *PpTCP18* may play an important role in peach branching. Yeast one-hybrid, electrophoretic mobility shift, dual-luciferase assays and *PpTCP18*-knockdown in peach leaf buds indicated that PpTCP18 could increase expression of *PpLBO1, PpMAX1*, and *PpMAX4*. Furthermore, transgenic Arabidopsis plants overexpressing *PpTCP18* clearly exhibited reduced primary rosette-leaf branches. Moreover, lncRNA sequencing and transient expression analysis revealed that lncRNA5 targeted *PpTCP18*, significantly increasing *PpTCP18* expression. These results provide insights into the mRNA and lncRNA network in the peach SL signaling pathway and indicate that PpTCP18, a transcription factor downstream of SL signaling, is involved in positive feedback regulation of SL biosynthesis. This role of PpTCP18 may represent a novel mechanism in peach branching regulation. Our study improves current understanding of the mechanisms underlying peach branching and provides theoretical support for genetic improvement of peach tree architecture.

## Introduction

Tree architecture is of great interest to plant breeders because it is a critical factor that affects the management of orchards, forests, and parks [1, 2]. In fruit crops, a proper balance between vegetative and reproductive growth is a key agronomic trait that has a great impact on fruit yield and quality [3]. Peach [*P. persica* (L.) Batsch] is the most economically important deciduous fruit crop in the world (FAO, http://faostat.fao.org); it exhibits excessive vegetative growth compared with other fruit trees, such as apple and pear. Reducing peach tree branching would reduce the need for pruning, decrease orchard management expenses, and increase fruit yield and quality [4]. Compared with standard peach trees, pillar peach trees require less pruning and are promising candidates for use in high-density peach production systems due to their narrow branch angles and fewer second-order branches [5]. However, the molecular mechanisms underlying peach branching are unclear. It is therefore important to explore the biological processes that yield low vegetative growth to allow breeding of peach cultivars that are conducive to labor-saving cultivation.

Tree architecture is mainly influenced by the spatial patterning of branches, which is determined by branching (branch number), branch angle, and internode length [1, 6–9]. Branching is controlled by a complex and interconnected regulatory network that responds to genetic, hormonal, and environmental factors [10, 11]. Decades ago, auxin was found to regulate meristem fate and was considered the major plant growth regulator (PGR) involved in shaping plant architecture by indirectly inhibiting branching [10, 12]. Exogenous auxin was also found to significantly inhibit branching [13].

The role of auxin in apical dominance was thought to be indirect [10]. Characterization of high-branching mutants in numerous species, including more axillary growth (*max*) in Arabidopsis, dwarf (*d*) in rice, and ramosus (*rms*) in pea revealed that the long-distance signaling molecule strigolactone (SL) is the key PGR that controls shoot branching [14–16]. The second messengers model posits that SLs are secondary messengers of auxin signaling [10]. SLs are synthesized through the β-carotene pathway via three oxidative reactions, which are carried out by a carotene isomerase (D27), two carotenoid cleavage dioxygenases (CCD7/MAX3 and CCD8/MAX4), and a cytochrome P450 (MAX1); bioactive SL or SL-like compounds are synthesized through multiple routes and involve in a range of enzymes including LATERAL BRANCHING OXIDOREDUCTASE (LBO) [17]. Bioactive SLs interact with the receptor protein D14/AtD14/DAD2/RMS3, a α/β-hydrolase [18, 19]. A complex is formed between SL and one of the three primary targets, the D53-like SUPPRESSOR OF MAX2 1 (SMAX1)-LIKE (SMXL) proteins D53/SMXL6,7,8. During perception, D14 and D53/SMXL6,7,8 are hydrolyzed by 26S proteasomes [20–22]. Mutations in SL biosynthesis and signaling genes, such as *OsMAX4, OsD14* [23], *OsD53* [24], and *AtMAX1* [25], increase branching/tillering by decreasing endogenous levels of SL or impacting SL signal transduction. This indicates that SL plays an important role in controlling branching/tillering.

Transcription factors in the TEOSINTE BRANCHED1/CYCLOIDEA/PROLIFERATING CELL FACTOR (TCP) family are downstream targets of SL signaling and are key factors involved in controlling branching. TCP family members, such as *TB1*, *BRC1*, and *FC1* [9–11, 14], contain a 59-residue homologous region named the TCP domain. BRC1 sub-family genes in Arabidopsis, rice, and pea are predominantly expressed in the axillary buds and are involved in bud dormancy; mutation of these genes increases branching/tillering [14, 26, 27]. Recently, these proteins were reported to be involved in a different pathway that controls branching. Brassinosteroid (BR) signaling protein (BES1) regulated branching by inhibiting *BRC1* expression through direct binding to the promoter and subsequent SMXL recruitment [28]. Likewise, SQUAMOSA PROMOTER BINDING PROTEIN-LIKE 14 (SPL14) influences branching by regulating expression of *OsTB1* through a GTAC *cis*-element in the promoter [29].

Long noncoding RNAs (lncRNAs), which are >200 nt and usually lack protein-coding potential, are transcribed by RNA polymerase II, III, or IV/V in plants [30]. LncRNAs contribute to regulation of gene expression by influencing DNA methylation, histone modification, and chromatin structure, and are potent *cis*- and *trans*-regulators of gene expression [31]. Although lncRNAs have been the focus of increased research interest in recent years, the field of lncRNA research is still in its infancy. This is especially true in plants, in which only a few lncRNAs have been studied and sufficiently described [32–35].

Although the functions of TCP genes have been characterized in model plants, the molecular mechanisms controlling branching/tillering have not been well characterized in woody plant, including peach. In this study, the mechanisms of PpTCP18 controls peach branching through regulating SL biosynthesis were investigated. GR24 treatment could obviously inhibit peach branching. PpTCP18 could regulate SL biosynthesis through a positive feedback mechanism by directly activating *PpLBO1*, *PpMAX3*, and *PpMAX4* expression. Furthermore, the expression of *PpTCP18* could be dramatically increased by lncRNA5. These results will shed light on how PpTCP18 may regulate peach branching at the molecular level.

## Results

### The pillar peach cultivar had fewer branches and higher SL content

**Figure 1:**
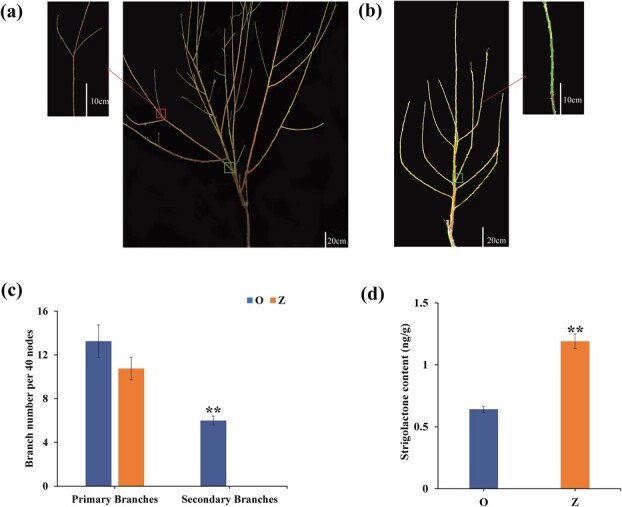
Branch number of standard [‘Okubo’ (O)] and pillar [‘Zhaoshouhong’ (Z)] peach trees. Representative images of (**a**) standard (‘O’) and (**b**) pillar (‘Z’) trees grown in an orchard for one year. The leaves were removed to visualize the architecture. Example primary branch nodes are outlined in green. Red arrows indicate enlarged images of example secondary branches. (**c**) Number of primary and secondary branches in ‘O’ and ‘Z’ plants within nodes 0 to 40. (**d**) Endogenous SL content in standard (‘O’) and pillar (‘Z’) peach trees. Values are the mean of three biological replicates, each containing three trees. ^**^P < 0.01 (Student’s *t*-test).

The number of branches within 40 nodes of ‘Okubo’ and ‘Zhaoshouhong’ were counted. Results indicated that ‘Okubo’ had slightly more primary branches than ‘Zhaoshouhong’ (Fig. 1a, b, and c). ‘Zhaoshouhong’ had almost no secondary branches; ‘Okubo’ had ~6.8 within the first 40 nodes (Fig. 1a, b, and c).

SL contents were measured in the leaf buds of ‘Zhaoshouhong’ and ‘Okubo’. The SL content was almost 2-fold higher in ‘Zhaoshouhong’ than ‘Okubo’ (Fig. 1d), suggesting that the lower branch number of ‘Zhaoshouhong’ may be related to the higher SL content.

### Exogenous strigolactone significantly decreased branch number in ‘Zhaoshouhong’

**Figure 2:**
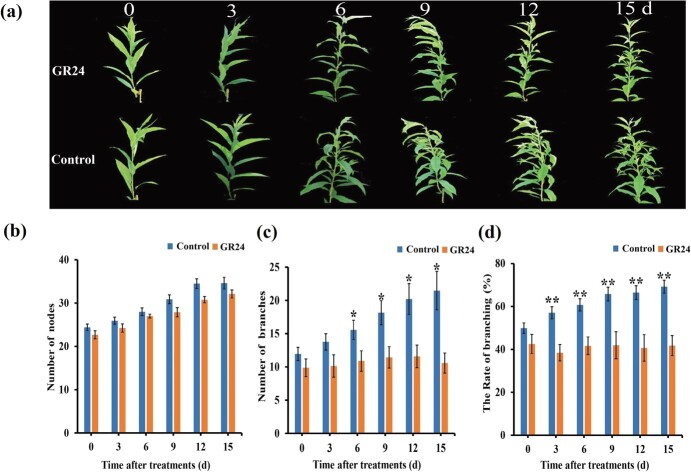
Exogenous GR24 treatment decreased peach branch number. (**a**) One-year-old ‘Zhaoshouhong’ plants were treated with the synthetic strigolactone GR24. Untreated ‘Zhaoshouhong’ plants were used as the control. **b–d** The number of nodes (**b**), branch number (**c**), and branching rate (**d**) were determined after GR24 treatment. The branching rate was calculated as the number of branches divided by the number of nodes. Values are the mean of three biological replicates (*n* = 5). ^*^P < 0.05, ^**^P < 0.01 (Student’s *t*-test).

Exogenous SL (GR24) treatment ‘Zhaoshouhong’ was conducted to verify the function of SL in controlling peach branching. The number of branches and nodes were observed for 15 d in control ‘Zhaoshouhong’ trees and those treated six times with GR24, respectively. Branching was largely inhibited in GR24-treated ‘Zhaoshouhong’ plants compared to the control (Fig. 2a, b, and c). By 9 d after treatment, the GR24-treated group had an average of 11.4 branches, whereas the control group had an average of 18.5 (Fig. 2c). The branching rate of GR24-treated ‘Zhaoshouhong’ plants was dramatically lower than control plants at 3 d after GR24 treatment (Fig. 2d), although the number of nodes did not significantly differ between control and GR24-treated plants (Fig. 2b and d). These results indicated that SL decreased the branch number of ‘Zhaoshouhong’ annual growth, and furthermore decreased the number of sylleptic branches.

### PpTCP18 and SL synthesis-related genes showed opposite expression patterns after GR24 treatment

**Figure 3:**
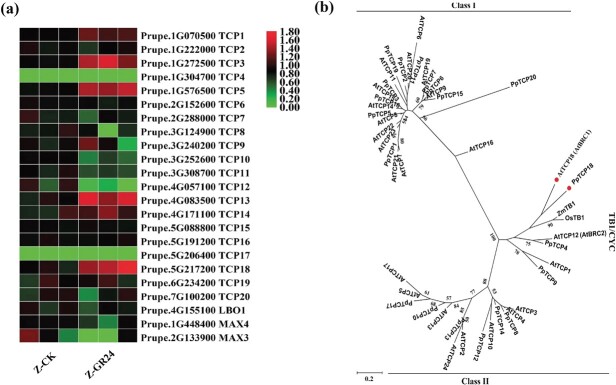
Expression profiles of TCPs and SL biosynthesis genes and phylogenetic analysis of PpTCPs. **a** Expression of TCP genes and SL biosynthesis genes in ‘Zhaoshouhong’ (‘Z’) after treatment with GR24 for 2 h. **b** Phylogenetic tree showing relationships between TCPs in *Prunus persica* (PpTCPs), *Arabidopsis thaliana* (AtTCPs), *Zea mays* (ZmTB1), and *Oryza sativa* (OsTB1). The tree was constructed using the maximum likelihood method.

To identify candidate genes involved in the SL pathway, two groups of transcriptome data were analysed, including ‘Zhaoshouhong’ and ‘Okubo’, the control and GR24 treated ‘Zhaoshouhong’. Exogenous GR24 treatment significantly inhibited the expression of SL synthesis-related genes such as *PpLBO1, PpMAX3*, and *PpMAX4* (Fig. 3a, bottom three rows). Transcriptome data from leaf buds sampled before bud break suggested that *PpLBO1, PpMAX3*, and *PpMAX4* were expressed at higher levels in ‘Zhaoshouhong’ than in ‘Okubo’ (Fig. S1, see online supplementary material). The expression profiles of SL-related genes were consistent with endogenous SL levels in ‘Zhaoshouhong’ and ‘Okubo’ (Fig. 1d).

TCP transcription factors are key factors involved in branching control [9–11]. To identify TCPs genes related to peach branching, 20 TCPs genes were identified in peach genome and further analyzed based on transcriptome data. The results suggested that in ‘Zhaoshouhong’, most *PpTCP*s were differentially expressed after exogenous GR24 treatment compared to the control (Fig. 3a). GR24 treatment caused upregulation of four of the 20 *PpTCP* genes: *PpTCP3, PpTCP5, PpTCP13,* and *PpTCP18* (Fig. 3a). Phylogenetic analysis of 20 PpTCPs, 24 AtTCPs, one *Zea mays* TCP (ZmTB1), and one *Oryza sativa* TCP (OsTB1) revealed two subclasses. Class I contained 13 AtTCPs and 11 PpTCPs; Class II contained 11 AtTCPs, 9 PpTCPs, ZmTB1, and OsTB1. Class II could be further subdivided into two groups: CIN and TB1/CYC. PpTCP18 belonged to the TB1/CYC group and was closely related to AtBRC1 (AtTCP18), which is the key factor that controls branching in Arabidopsis [14] (Fig. 3b). PpTCP18 was therefore selected for further study. Additional transcriptome data from untreated leaf buds showed that *PpTCP18, PpLBO1, PpMAX3*, and *PpMAX4* were expressed at higher levels in ‘Zhaoshouhong’ than in ‘Okubo’ (Fig. S1, see online supplementary material). These results suggested that PpTCP18 may participate in peach tree branching by regulating SL biosynthesis.

### PpTCP18 was localized to the nucleus and cell membrane

A PpTCP18:GFP fusion construct was generated and expressed in tobacco leaves. At three days after injection, the fluorescent signal of the PpTCP18:GFP fusion protein was exclusively localized to the nucleus and cell membrane (Fig. S2, see online supplementary material). In contrast, the fluorescent signal of the GFP control was distributed throughout the entire cell (Fig. S2, see online supplementary material).

### PpTCP18 directly interacted with the promoters of *PpLBO1, PpMAX3,* and *PpMAX4* to enhance their expression

**Figure 4:**
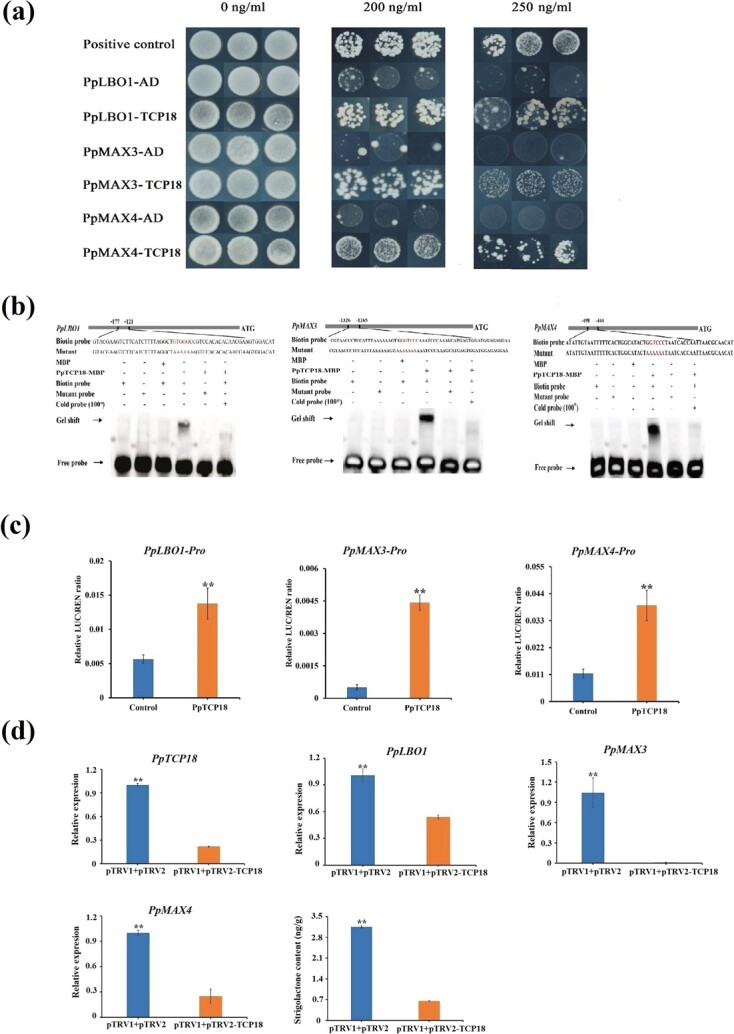
PpTCP18 enhanced *PpLBO1*, *PpMAX3*, and *PpMAX4* transcription. **a** Yeast one-hybrid (Y1H) analysis showed that PpTCP18 bound the *PpLBO1*, *PpMAX3*, and *PpMAX4* promoters. Aureobasidin A (AbA) (200 and 250 ng/ml) was used for screening. Rec-P53 and the P53-promoter were the positive controls. Promoters driving the empty vector were used as negative controls. **b** Electrophoretic mobility shift assays (EMSAs) showed that PpTCP18 bound the GGNCCC/GGGNCC motif in the *PpLBO1*, *PpMAX3*, and *PpMAX4* promoters. **c** Dual luciferase assays indicated that PpTCP18 activated transcription of *PpLBO1, PpMAX3*, and *PpMAX4*. Plasmids containing PpTCP18 and either PpLBO1, PpMAX3, or PpMAX4 were introduced into tobacco leaves *via Agrobacterium tumefaciens*-mediated transformation to assess the activation of *PpLBO1*, *PpMAX3*, and *PpMAX4* promoters by PpTCP18. Firefly luciferase (LUC)/Renilla luciferase (REN) ratios were significantly higher when leaves were co-transformed with the PpTCP18 vector than with the empty control vector, suggesting that PpTCP18 enhanced *PpLBO1*, *PpMAX3*, and *PpMAX4* promoter activity. **d** Virus-induced gene silencing of *PpTCP18* in peach leaf buds reduced expression of *PpLBO1*, *PpMAX3*, and *PpMAX4*, and reduced the endogenous SL concentration. Transcript levels are shown for *PpTCP18*, *PpLBO1*, *PpMAX3*, and *PpMAX4* in peach leaf buds infiltrated with the empty (control) or TRV2:PpTCP18 vector. Values shown are the mean ± standard deviation of three biological replicates. ^**^P < 0.01 vs. control (Student’s *t*-test).

Transcriptome analysis of ‘Okubo’ and ‘Zhaoshouhong’ leaf buds (before bud break) showed that *PpLBO1*, *PpMAX3*, and *PpMAX4* had similar expression patterns to that of *PpTCP18* (Fig. S1, see online supplementary material), indicating that PpTCP18 may regulate the expression of these SL biosynthesis genes. To verify this hypothesis, a bioinformatics analysis was performed on the promoter sequences of *PpLBO1*, *PpMAX3*, and *PpMAX4*. A TCP binding motif (GGNCCC/GGGNCC) was found in each promoter (Table S1, see online supplementary material). A yeast one-hybrid (Y1H) experiment was carried out to verify the interactions of PpTCP18 with the *PpLBO1*, *PpMAX3*, and *PpMAX4* promoters. When PpTCP18 was co-transformed in yeast cells with a reporter construct containing either the *PpLBO1, PpMAX3*, or *PpMAX4* promoter, the yeast grew well in the presence of Aureobasidin A (AbA) (200 ng/ml). When they were co-transformed with the empty vector pGADT7, the yeast did not grow well on the same medium (Fig. 4a). EMSAs were performed to further confirm the interactions between PpTCP18 and the *PpLBO1*, *PpMAX3*, and *PpMAX4* promoters. The coding sequence (CDS) of *PpTCP18* was cloned into the pGEX-6P-1 vector, and recombinant PpTCP18:MBP protein was purified (Fig. S3, see online supplementary material). EMSA results suggested that PpTCP18 could directly bind the probes derived from the *PpLBO1*, *PpMAX3*, and *PpMAX4* promoters via the GGNCCC/GGGNCC motif. The band shifts observed for PpTCP18 incubated with the *PpLBO1*, *PpMAX3*, or *PpMAX4* promoters were greatly weakened by the addition of 100-fold unlabeled probes, and disappeared entirely when the GGNCCC/GGGNCC motif was mutated to a polyA sequence (Fig. 4b). These results indicated that PpTCP18 could directly bind to the SL-responsive elements located in the *PpLBO1*, *PpMAX3*, and *PpMAX4* promoters via the GGNCCC/GGGNCC motif.

To investigate whether PpTCP18 could affect the activity of SL biosynthesis-related genes, the promoters of *PpLBO1*, *PpMAX3*, *PpMAX4* were cloned into dual luciferase reporter vector pGreenII 0800-LUC, respectively. Then either of them and 35:PpTCP18 were transiently expressed in tobacco leaves. Results showed that transient infiltration of tobacco leaves with *Agrobacterium* carrying the 35S:PpTCP18 vector enhanced the activity of the *PpLBO1*, *PpMAX3*, and *PpMAX4* promoters by 2.5-, 8.7-, 3.4-fold compared with the empty vector (Fig. 4c). To further verify the transcriptional activation ability of *PpTCP18*, a virus-induced gene silencing (VIGS) experiment was performed to knock down *PpTCP18* in peach. The results suggested that transient *Agrobacterium* infiltration ofpillar peach leaf buds with the TRV2:PpTCP18 vector reduced expression of *PpLBO1*, *PpMAX3*, and *PpMAX4*, by 1.9-, 94.9-, and 4-fold, respectively, compared to the control group infiltrated with empty TRV2 vector. Endogenous SL levels in plants treated with PpTCP18 and untreated controls were 0.65 and 3.15 ng/g, respectively (Fig. 4d). Collectively, these results demonstrated that PpTCP18 could directly bind to the *PpLBO1*, *PpMAX3*, and *PpMAX4* promoters and significantly enhance their expression.

### PpTCP18 inhibited branching in Arabidopsis

In order to verify the function of PpTCP18 in branching, *PpTCP18* overexpression vector was constructed and stably transformed into Arabidopsis. Three transgenic *PpTCP18*-overexpression lines (TCP18–1, TCP18–3, and TCP18–5) that had higher *PpTCP18* transcript levels than the WT were selected for further analysis (Fig. S4, see online supplementary material). After two weeks in a growth chamber, the rosette leaves were counted. Results showed that PpTCP18 overexpression significantly decreased the number of rosette leaves in the transgenic lines; there were an average of 16.25, 15.67, and 15.75 leaves for PpTCP18–1, PpTCP18–3, and PpTCP18–5, respectively, compared to 24 leaves in the WT (Fig. S5a and b, see online supplementary material). There were no obvious differences in the length and width of the rosette leaves between the transgenic lines and the WT (Fig. S5c and d, see online supplementary material). The primary cauline leaf branches (CI) and primary rosette leaf branches (RI) (Fig. S5e, see online supplementary material) were counted two weeks after flowering. *PpTCP18* overexpression led to significantly fewer primary rosette-leaf branches, with RI values of 5.66, 5.58, 5.41, and 9.00 for PpTCP18–1, PpTCP18–3, PpTCP18–5, and WT, respectively (Fig. S5f and g, see online supplementary material). There were no significant differences in the CI between the transgenic lines and WT (Fig. S5h, see online supplementary material). The endogenous SL content was higher in the nodes of transgenic plants compared to the WT. Consistent with the measured SL concentrations (Fig. S5i, see online supplementary material), expression levels of SL-related genes (namely *AtMAX2, AtMAX4, AtBRC1*, and *AtBRC2*) were significantly higher in transgenic lines compared with WT; furthermore, *SMXL6/7/8* expression was markedly decreased in transgenic lines, whereas there were no differences in *AtBES1* expression (Fig. S5j, see online supplementary material). ABA biosynthesis-related genes (*AtNCED1/2/3/4*) were upregulated in transgenic lines compared to WT, but *AtNCED5/6* transcripts could not be detected, perhaps due to low expression levels (Fig. S5I, see online supplementary material). These results suggested that PpTCP18 may prevent primary rosette branching in Arabidopsis by influencing the SL and ABA pathways.

### LncRNA5 (TCONS_00066816) increased *PpTCP18* expression

**Figure 5:**
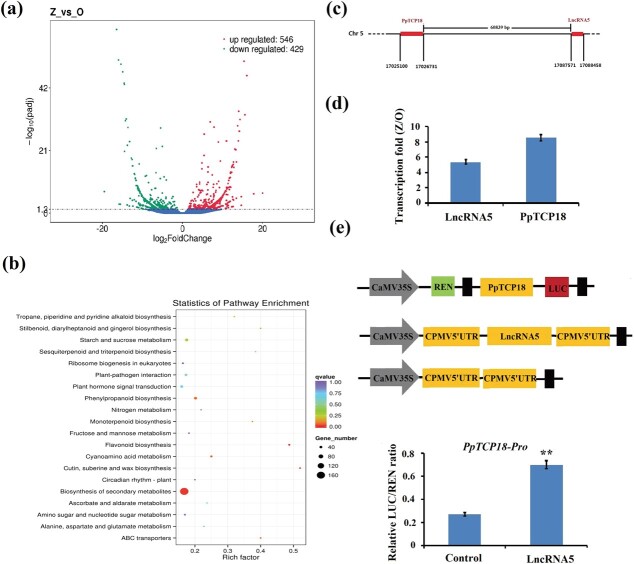
LncRNA increased *PpTCP18* expression*.***a** Volcano plot showing differential lncRNA expression between ‘Okuba’ (‘O’) and ‘Zhaoshouhong’ (‘Z’). **b** KEGG pathway enrichment analysis of genes targeted by differentially expressed lncRNAs. **c** LncRNA5 is located downstream of *PpTCP18*. **d** Relative *PpTCP18* and lncRNA5 expression in Z/O. **e** A dual luciferase analysis showed that lncRNA5 activated *PpTCP18* transcription. Values are the mean ± standard deviation of three biological replicates. ^**^P < 0.01 vs. control (Student’s *t*-test).

To select lncRNA targeted *PpTCP18* and verify whether the candidate lncRNA could regulate the expression of *PpTCP18*. LncRNA libraries were constructed from ‘Okubo’ and ‘Zhaoshouhong’ leaf buds before bud break. In total, 14 503 lncRNAs were identified in peach, 975 of which were differentially expressed between ‘Okuba’ and ‘Zhaoshouhong’. There were 546 upregulated and 429 downregulated lncRNAs in ‘Zhaoshouhong’ compared to ‘Okuba’ (Fig. 5a), with the former including TCONS_00066816 (lncRNA5). The Kyoto Encyclopedia of Genes and Genomes (KEGG) suggested that the genes targeted by differentially expressed lncRNAs were involved in a variety of biological processes, including starch and sucrose metabolism, flavonoid biosynthesis, and hormone responses (Fig. 5b). Target gene prediction results showed that *PpTCP18* was the target of lncRNA5. LncRNA5, located 60 839 bp downstream of *PpTCP18* (Fig. 5c), showed a similar expression pattern with that of *PpTCP18*; lncRNA5 was expressed 5-fold higher in ‘Zhaoshouhong’ than ‘Okuba’ (Fig. 5d). The *PpTCP18* promoter was cloned into the dual-luciferase reporter pGreenII 0800-LUC vector to determine whether the expression of *PpTCP18* could be regulated by lncRNA5 (Fig. 5e). Dual luciferase assay (DLR) results showed that *PpTCP18* promoter activity was significantly increased by lncRNA5 (Fig. 5e). These results suggested that lncRNA5 could increase *PpTCP18* expression.

## Discussion

Pillar peach trees require less pruning, which can greatly reduce costs [5]. In this study, the pillar cultivar ‘Zhaoshouhong’ and the standard cultivar ‘Okubo’ were compared. ‘Zhaoshouhong’ had the same number of nodes as ‘Okubo’ but fewer secondary branches. This finding was similar to results reported by Bassi and Dima showing fewer second-order branches of columnar (pillar) peach trees compared to standard peach trees [5]. The endogenous SL content was significantly higher in ‘Zhaoshouhong’ than in ‘Okubo’, and GR24 treatment significantly inhibited peach branching. These results indicated that SL plays an important role in apical dominance in peach [36], and that the SL level was closely negatively correlated with branch number.

*PpTCP18* expression was negatively correlated with branch number in the pillar cultivar ‘Zhaoshouhong’ compared to the standard cultivar ‘Okubo’. The TCP protein BRC1 is also reportedly upregulated by GR24 treatment in other species [27, 37], whereas the SL biosynthesis genes *LBO1, MAX3*, and *MAX4* were downregulated by GR24 treatment in peach and maize [37]. *PpTCP18* levels were positively correlated with SL content, and silencing *PpTCP18* in peach leaf buds significantly decreased endogenous SL content, and the SL content in transgenic Arabidopsis lines overexpressing *PpTCP18* was increased. Because *AtMAX2, AtMAX4, AtBRC1*, *AtBRC2*, *AtSMXL6, AtSMXL7*, and *AtSMXL8* play important roles in branching regulation, the loss-of-function mutant lines *atmax2, atmax4, atbrc1*, and *atbrc2* show significantly higher branch numbers [14, 38–40]. AtSMXL6, AtSMXL7, and AtSMXL8 act as putative transcriptional repressors in the SL pathway; after SL induces degradation of AtSMXL6, 7, and 8, the downstream targets of AtSMXLs (including AtBRC1) are de-repressed, preventing branching [17, 41–43]. We therefore measured expression of these genes and found that *AtMAX2, AtMAX4, AtBRC1*, and *AtBRC2* were greatly upregulated in plant lines overexpressing *PpTCP18*; while *AtSMXL6, AtSMXL7*, and *AtSMXL8* were downregulated in *PpTCP18* overexpression lines. SLs may inhibit branching in part by inducing ABA biosynthesis [44] through upregulating the ABA biosynthesis genes *AtNCED1/2/3/4*. These results suggested that PpTCP18 may inhibit Arabidopsis branching by influencing the SL and ABA signaling pathways.

LncRNAs coordinate gene expression via chromatin modification, acting either as endogenous target mimics (eTMs) or through *cis*- or *trans*-activation [30, 31]. Studies have demonstrated that lncRNAs participate in regulating fruit ripening [33], drought tolerance [45], disease resistance [46], and fruit coloring [35]. However, there have been few reports of lncRNA involvement in the SL signaling pathway. Here, we found that lncRNA5 in peach (TCONS_00066816) upregulated *PpTCP18*. LncRNA5 may enhance *PpTCP18* expression through recognition of the *PpTCP18* sequence through sequence complementarity and R-loop formation; this would be comparable to a regulatory mechanism in apple (*Malus domestica*), in which MdLNC610 increases *MdACO1* expression [35].

Previous studies have suggested that SL may be the auxin secondary messenger that directly inhibits plant branching [10, 47]; that SL biosynthesis is subject to positive feedback regulation by auxin, whereas auxin biosynthesis is subject to negative feedback regulation by SL [38]; and that ABA biosynthesis is induced by SL [48]. In contrast to these previous studies, we here identified a novel mechanism involved in peach branch number regulation; PpTCP18 controls peach branching by positive feedback regulation of SL biosynthesis. A hypothesis was proposed to explain the role of PpTCP18 in peach branching: (i) in standard peach, the endogenous SL content was very low, leading to lower *PpTCP18* expression and more the branch number; and (ii) in pillar peach, the SL concentration was higher, inducing *PpTCP18* expression, which in turn positively regulated SL biosynthesis gene expression and SL content. Furthermore, *PpTCP18* expression was dramatically increased by lncRNA5, greatly inhibiting branch number in pillar peach trees.

## Materials and methods

### Plant materials

Representatives of two types of peach tree, ‘Okubo’ (‘O’, standard peach) and ‘Zhaoshouhong’ (‘Z’, pillar peach), were grown in the research orchard of the Henan Agricultural University in Zhengzhou, China. Phenotypic analyses of ‘Okubo’ and ‘Zhaoshouhong’ annual growth were conducted in May 2019, with leaves removed to assess branch number. Leaf buds were sampled from both cultivars in April (before bud break) for SL measurements and transcriptome analyses. Annual growth of ‘Zhaoshouhong’ was used for GR24 treatment, and the phloem was sampled at branch junctions for transcriptome analysis. Leaf buds after bud break (3–4 cm) were sampled from three-year-old ‘Zhaoshouhong’ trees and used in virus-induced gene silencing (VIGS) experiments. Arabidopsis (Col-0) was used for stable transformation. *Nicotiana benthamiana* was used for dual-luciferase assays (DLR).

### SL measurements

SL levels were measured by NJRuiYuan Co., Ltd. as described by Ruiz-Lozano [49]. Peach leaf buds (5 g) were ground into powder with liquid nitrogen. The powder was extracted with 0.5 mL 40% acetone (v/v), followed by vortexing for 2 min, then centrifugation at 8000 × g for 5 min. The pellets were collected and extracted twice with 0.5 mL 50% acetone (v/v). The supernatant was purified with membrane filters (0.22 μm pore size). SL content was then measured by HPLC-MS/MS.

### TCP gene family identification and phylogenetic tree construction

To identify *TCP* genes in peach, sequences of Arabidopsis *TCP* genes were downloaded from PlantTFDB (v4.0) [50] and used as queries in BLAST searches against the peach genome. Twenty *PpTCP* genes were identified in peach and named *PpTCP1* through *PpTCP20* based on their chromosome positions. Amino acid sequences of the TCPs from *P. persica* (PpTCPs) and *Arabidopsis thaliana* (AtTCPs) [14] were used together with ZmTB1 [51] from *Z. mays* and OsTB1 [52] from *O. sativa* (Table S2, see online supplementary material) to construct a neighbor-joining phylogenetic tree using MEGA 5.0 [50]. The maximum likelihood method was employed with 1000 bootstrap replicates.

### Exogenous GR24 treatment

The synthetic SL GR24 (Coolaber, Beijing, China) was dissolved in acetone, then diluted with water to a final concentration of 20 μM. This solution was used to treat the annual growth of ‘Zhaoshouhong’. Water was used as the control. Plants were treated every 3 d for a total of six treatments. The node number and branch number were counted every 3 d, and the branching rate was calculated as branch number divided by node number. Each treatment was conducted with three biological replicates, and each replicate contained five annual growth plants of ‘Zhaoshouhong’.

To identify genes that respond to GR24, the whole-plant annual growth of ‘Zhaoshouhong’ was treated with GR24. The branch junctions of treated and control plants were sampled after treatment for 0 and 2 h, then stored in a −80°C freezer prior to transcriptome analysis.

### RNA extraction, cDNA synthesis, and gene expression analysis

RNA extraction, cDNA synthesis, and quantitative reverse transcription (qRT)-PCR experiments were performed as described by Wang *et al.* [53]. RNA was extracted from peach buds sampled in April using a Total RNA Kit (Tiangen, Beijing, China). Purified RNA was used for transcriptome analysis and qRT-PCR. cDNA synthesis was performed using the FastKing RT Kit (Tiangen, Beijing, China). Relative expression levels of *PpTCP18*, *PpLBO1, PpMAX3*, and *PpMAX4* were analysed with qRT-PCR. Actin (*Prupe.1G234000*) was used as the internal control for normalizing gene expression. There were three independent biological replicates. Primers are shown in Table S3 (see online supplementary material).

Heatmap analysis was performed on the transcriptome data using the CLUSTER software package [50] and Java Treeview [50]. The reads per kilobase of transcript per million mapped reads (RPKM) values of *PpLBO1 (Prupe.4G155100), PpMAX3 (Prupe.1G448400), PpMAX4 (Prupe.2G133900)*, and the *PpTCP* genes used in the heatmap analysis are shown in Table S4 (see online supplementary material).

### Gene cloning and promoter analysis

The *PpTCP18* gene and the 2 kb promoter regions upstream of the *PpLBO1*, *PpMAX3*, and *PpMAX4* start codons were cloned as described by Wang *et al.* [53]. Primers are shown in Table S3 (see online supplementary material). The online tool plantCARE (http://bioinformatics.psb.ugent.be/webtools/plantcare/html/) was used to identify GGNCCC/GGGNCC motifs.

### Subcellular localization of PpTCP18

The CDS of *PpTCP18* was cloned into the pSAK277-GFP vector to construct the PpTCP18:GFP fusion protein driven by the CaMV 35S promoter. A Leica SP8 Confocal Microscope (Wetzlar, Germany) was used to observe fluorescent signals. mCherry and Cd3–1009 [54] were the nuclear and membrane-localized marker genes used, respectively. Primers are shown in Table S4 (see online supplementary material).

### Virus-induced gene silencing (VIGS)

Transient gene expression was performed as described by Liu *et al.* [55]. Approximately 200 bp of the non-conserved complementary sequence of *PpTCP18* was amplified and used for gene silencing. Primers are shown in Table S4 (see online supplementary material). The PCR product was purified and cloned into the TRV2 vector. Plasmids were transformed into *Agrobacterium tumefaciens* strain GV3101. Peach buds (3–4 cm) from ‘Zhaoshouhong’ were soaked in 75% alcohol for 1 min and washed three times with sterile water. The sterilized peach leaf buds were pre-cultured on Murashige and Skoog (MS) medium for 1 d, then submerged in a mixture of *A. tumefaciens* strains carrying TRV2 and TRV1 vectors and vacuum infiltrated (−70 kPa). Peach leaf buds were washed three times in sterile water, then cultured in MS medium for 2 d. Finally, leaf buds were frozen in liquid nitrogen and stored at −80°C prior to qRT-PCR analysis and SL measurements. There were three independent replicates of these experiments.

### Yeast one-hybrid (Y1H)

The CDS of *PpTCP18* was cloned into the pGADT7 vector. The promoter regions ~2 kb upstream of the *PpLBO1*, *PpMAX3*, and *PpMAX4* start codons were amplified and ligated into the pAbAi vector. A Matchmaker™ Gold Yeast One-Hybrid Library Screening System Kit (Clontech, San Francisco, CA, USA) was used to test interactions between PpTCP18 and the promoters. Primers are shown in Table S3 (see online supplementary material).

### Dual-luciferase assay (DLR)

The promoter sequences of *PpLBO1, PpMAX3, PpMAX4*, and *PpTCP18* were cloned into the pGreenII 0800-LUC vector. LncRNA5 and the CDS of *PpTCP18* were cloned separately into the pSAK277 vector. The LUC and REN luciferase activity levels were measured using a DLR kit (Promega, Madison, WI, USA) following the manufacturer’s instructions. For each assay, at least six measurements were obtained. Primers are listed in Table S3 (see online supplementary material).

### Electrophoretic mobility shift assay (EMSA)

The CDS of *PpTCP18* was cloned into the pMAL-c5x vector containing the MBP tag, then the plasmid was transformed into *Escherichia coli*. Recombinant PpTCP18–MBP protein was expressed and purified as described by Wang *et al.* [53]. An EMSA Kit (Thermo Scientific, Waltham, MA, USA) was used following the manufacturer’s instructions. Sequences containing the GGNCCC/GGGNCC motifs derived from the *PpLBO1, PpMAX3*, and *PpMAX4* promoters (Table S5, see online supplementary material) were labeled with biotin at the 5′ termini and were used as probes. The same DNA fragments without labels were used as competitors (cold probes). DNA fragments with the GGNCCC/GGGNCC motif changed to a polyA sequence were used as mutated probes.

### Plant transformation and phenotype analysis

Arabidopsis plants were transformed with the pSAK277-PpTCP18 construct using an *Agrobacterium*-mediated transformation protocol as described by Zhang *et al.* [56]. Arabidopsis seeds of the T_2_ generation were sown on commercial soil after cold treatment at 4°C for 3 d. Plants were transferred to a growth chamber at 20°C with a 16/8 h light/dark cycle. Leaves were counted at 2 weeks after transfer to the growth chamber. Primary-leaf branches (branches >0.5 cm long) were counted after the main inflorescence was visible. Each plant line was represented by three biological replicates; each replicate contained three individual T_2_ plants. For SL content measurement and SL-related gene expression analysis, only the nodes were sampled. All gene expression analyses were performed with three independent biological replicates. Primers are shown in Table S3 (see online supplementary material).

### LncRNA sequencing and bioinformatic analysis

Leaf buds from ‘Okubo’ (O-1, O-2, O-3) and ‘Zhaoshouhong’ (Z-1, Z-2, Z-3) were sampled in April (before bud break) and sent to Novogene (Beijing, China) for lncRNA library construction. Differentially expressed lncRNAs were identified using DESeq [2] software. Several databases were used to predict genes targeted by differentially expressed lncRNAs, including Volcano plot and the Kyoto Encyclopedia of Genes and Genomes (KEGG). LncRNA5 was selected and analysed with a DLR assay.

### Statistical analysis

Data were analysed with two-tailed Student’s *t*-tests. Microsoft Excel 2010 was used for statistical analyses. Statistical test parameters and statistical significance are noted in the figure legends.

## Acknowledgments

We thank Anita K. Snyder for copyediting the manuscript. This work was supported by the National Key Research and Development Program of China (2019YFD1000100), the Henan Province Outstanding Foreign Scholar Program (GZS2020007), the Modern Agricultural Industry Technology Systems Project of Henan Province (HARS-22-09-G1), the Special Fund of Henan Province for Agro-scientific Research in the Public Interest (No. 201300110500), the Youth Fund of Henan Province (No. 30602313), and the Innovation Fund of Henan Agricultural University (No. 30500873).

## Author contributions

Conceptualization: B.T. and J.F.; methodology development: Q.W.; bioinformatic data analyses: H.Z. and X.L.; experimental validation:L.Y., and Y.H.; formal analysis: J.C., W.W., and L.L.Z.; investigation: Q.W.; resources: X.Z., X.Y., and J.L.; data curation: X.W.; writing – original draft preparation: X.W.; writing – review and editing: B.T.; visualization: B.T.; supervision: J.F.; project administration: B.T. and J.F.; funding acquisition: X.W., B.T., and J.F. All authors have read and agreed to the published version of the manuscript.

## Data availability

All data needed to evaluate the conclusions stated in this paper are present in the paper and/or the supplementary materials.

## Conflict of interest

The authors declare that they have no conflicts of interest related to this work.

## Supplementary data

Supplementary data is available at *Horticulture Research* online.

## Supplementary Material

Web_Material_uhac224
