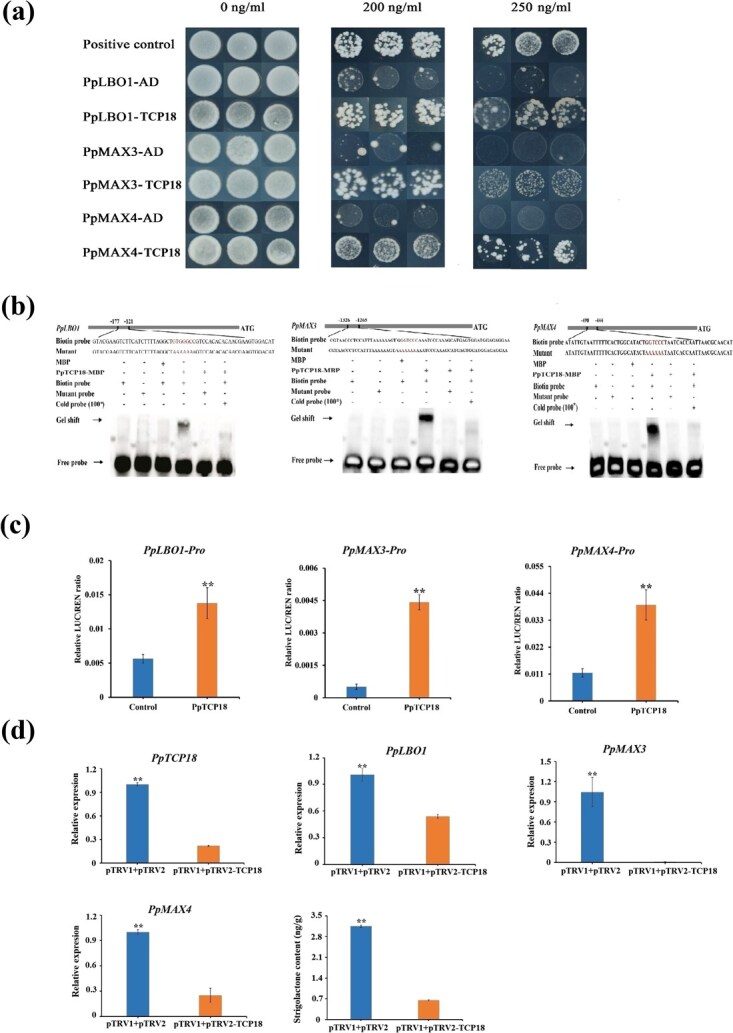# Correction to: *PpTCP18* is upregulated by lncRNA5 and controls branch number in peach (*Prunus persica*) through positive feedback regulation of strigolactone biosynthesis

**DOI:** 10.1093/hr/uhaf139

**Published:** 2025-05-30

This is a correction to: Xiaobei Wang, Qiuping Wang, Lixia Yan, Yuhang Hao, Xiaodong Lian, Haipeng Zhang, Xianbo Zheng, Jun Cheng, Wei Wang, Langlang Zhang, Xia Ye, Jidong Li, Bin Tan, Jiancan Feng, *PpTCP18* is upregulated by lncRNA5 and controls branch number in peach (*Prunus persica*) through positive feedback regulation of strigolactone biosynthesis, *Horticulture Research*, Volume 10, Issue 1, January 2023, uhac224, https://doi.org/10.1093/hr/uhac224.

Since the publication of this article, the Editors noticed errors within Figure 4A in the originally published version of this manuscript. Three plaques were wrong due to an oversight. They are negative control PpMAX4-AD (6th row, panel 1 and 4) and the positive control (1st row, panel 8) control. 

The editors have affirmed that this error does not affect the results or conclusions of the article. The authors would like to apologize for this error and are now issuing a correction for Figure 4A.

Figure 4A should read:

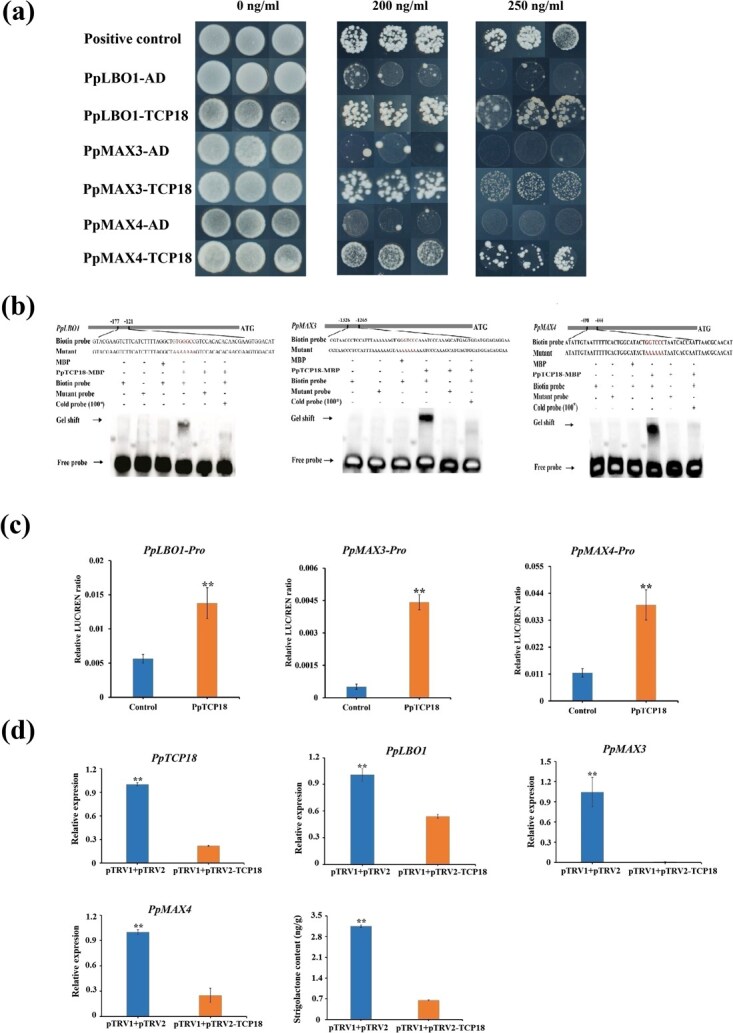



instead of: