# Correction to: Discovery of bifunctional diterpene cyclases/synthases in bacteria supports a bacterial origin for the plant terpene synthase gene family

**DOI:** 10.1093/hr/uhaf170

**Published:** 2025-07-28

This is a correction to: Xinlu Chen, Meimei Xu, Jin Han, Mark Schmidt-Dannert, Reuben J. Peters, Feng Chen, Discovery of bifunctional diterpene cyclases/synthases in bacteria supports a bacterial origin for the plant terpene synthase gene family, Horticulture Research, Volume 11, Issue 10, October 2024, uhae221, https://doi.org/10.1093/hr/uhae221.

In the originally published version of this manuscript, our assignment of the product for the fused diterpene cylase–synthase (DCS) from Streptomyces sp. GS7 (StrDCS) was incorrect. Rather than syn-abieta-11,13(15)-diene, this is actually syn-abieta-7,13-diene, as first indicated by personal communication from Prof. Jeffrey Rudolf (Univ. Florida), based up on his investigations of a very closely related DCS (1), and supported by computational NMR shift prediction (2). Specifically, using the CHESHIRE CCAT webtool (http://cheshirenmr.info/index.htm), which revealed substantially greater divergence between predicted and observed chemical shifts for the previous double-bond isomer of syn-abietadiene [i.e., 11,13(15)] versus current [i.e., 7,13]. As a consequence, Figure 3 in this publication should be replaced by that shown here immediately below.

Figure 3. Bacterial DCS activity. Genes were recombinantly expressed in *E. coli* also engineered to produce GGPP, and enzymatic products extracted from the resulting induced cultures for analysis by GC-MS. Shown here are chromatograms and schemes indicating the relevant reactions for A) CseDCS, B) ChjDCS, and C) StrDCS.


