# Supplementary material for: PpTCP18 is upregulated by lncRNA5 and controls branch number in peach (Prunus persica) through positive feedback regulation of strigolactone biosynthesis
Source: Hortic Res. 2022 Oct 7;10(1):uhac224. doi: 10.1093/hr/uhac224 (PMC9832876; doi:10.1093/hr/uhac224)
Supplement: Web_Material_uhac224 [file web_material_uhac224.zip › Fig. S1.docx]

(**b**)

(**a**)


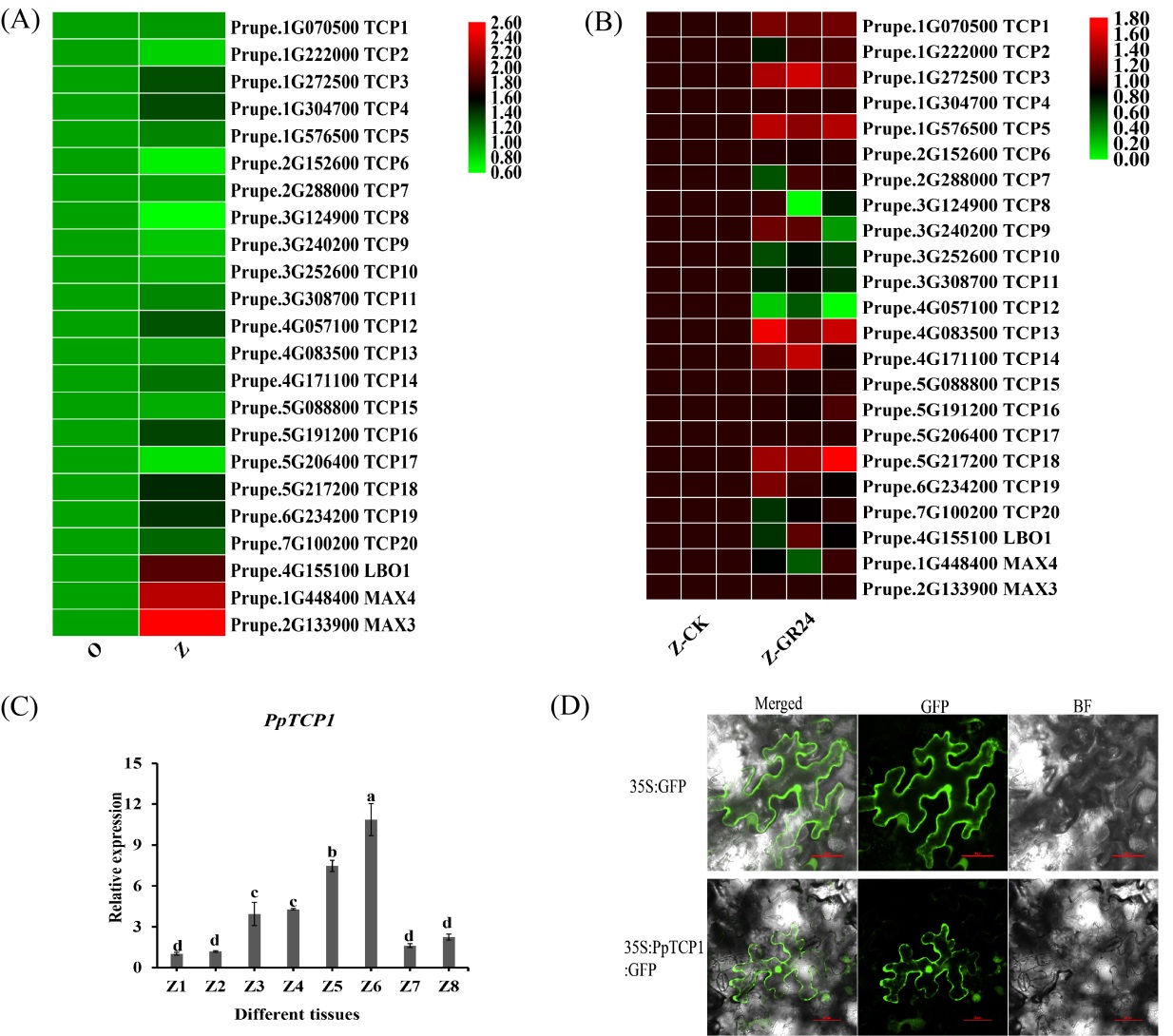

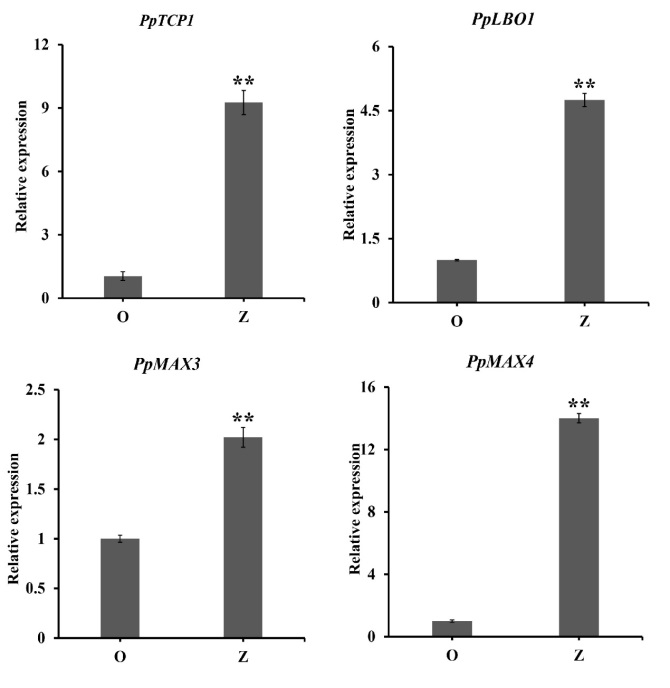


***PpTCP18***

**Figure S1**. The expression profiles of SL-related genes. (**a**) Heatmap analysis of TCPs and SL biosynthesis genes in ‘Zhaoshouhong’ (‘Z’) and ‘Okubo’ (‘O’) peach. (**b**) qRT-PCR analysis of *PpTCP18*, *PpLBO1*, *PpMAX3* and *PpMAX4* (** represent significance at P <0.01).
