# Supplementary material for: PpTCP18 is upregulated by lncRNA5 and controls branch number in peach (Prunus persica) through positive feedback regulation of strigolactone biosynthesis
Source: Hortic Res. 2022 Oct 7;10(1):uhac224. doi: 10.1093/hr/uhac224 (PMC9832876; doi:10.1093/hr/uhac224)
Supplement: Web_Material_uhac224 [file web_material_uhac224.zip › Fig. S2.docx]

**
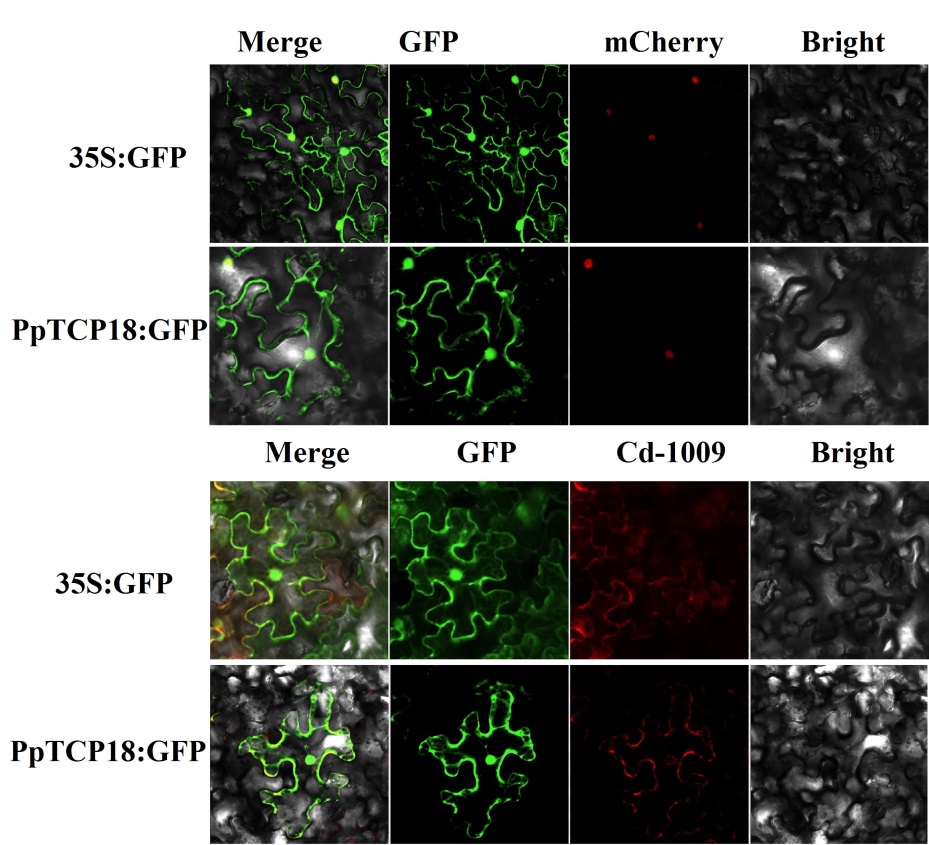
**

**Figure S2**. Subcellular localization of PpTCP18. mCherry and Cd3-1009 were used as nuclear and membrane-localized marker genes, respectively.
