# Supplementary material for: PpTCP18 is upregulated by lncRNA5 and controls branch number in peach (Prunus persica) through positive feedback regulation of strigolactone biosynthesis
Source: Hortic Res. 2022 Oct 7;10(1):uhac224. doi: 10.1093/hr/uhac224 (PMC9832876; doi:10.1093/hr/uhac224)
Supplement: Web_Material_uhac224 [file web_material_uhac224.zip › Fig. S3.docx]

1 2


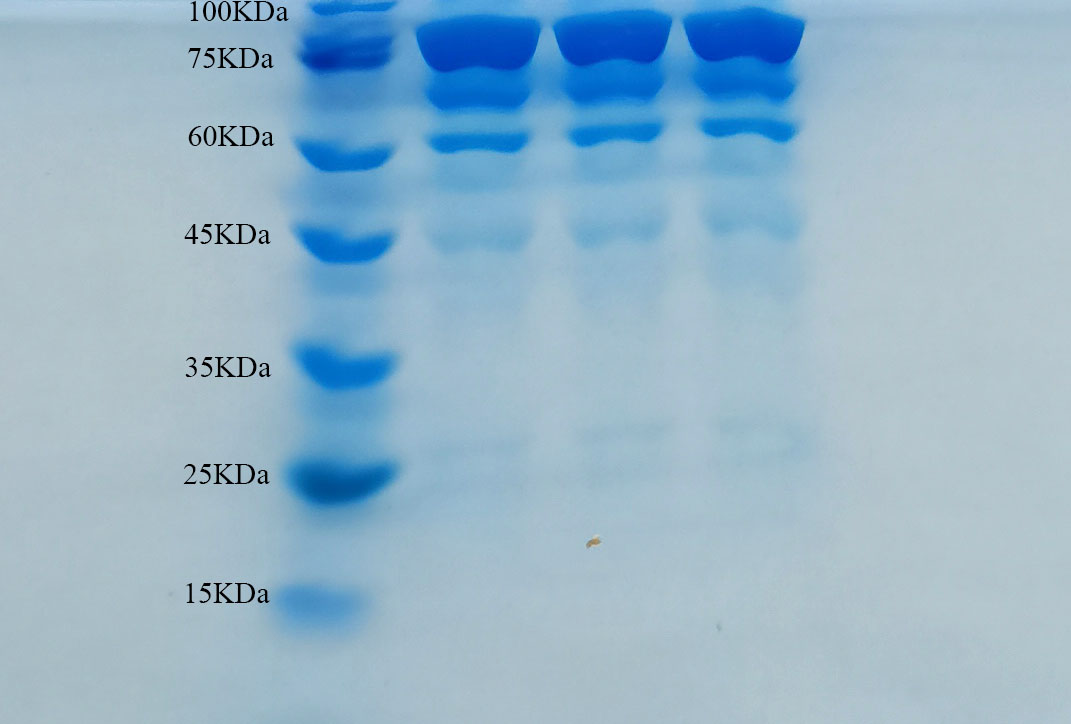


PpTCP18-MBP

**Figure S3.** Purified protein used for EMSA assay. The recombinant PpTCP18-MBP protein was purified and separated in an SDS-PAGE gel that was then stained with Coomassie blue. 1: Marker; 2: After purification. Per lane loading 5 µg purified protein.
