# Supplementary material for: PpTCP18 is upregulated by lncRNA5 and controls branch number in peach (Prunus persica) through positive feedback regulation of strigolactone biosynthesis
Source: Hortic Res. 2022 Oct 7;10(1):uhac224. doi: 10.1093/hr/uhac224 (PMC9832876; doi:10.1093/hr/uhac224)
Supplement: Web_Material_uhac224 [file web_material_uhac224.zip › Fig. S4.docx]

**M TCP18-1 TCP18-2 TCP18-3 TCP18-4 TCP18-5 TCP18-6 Plasmid Control**

(**a**)


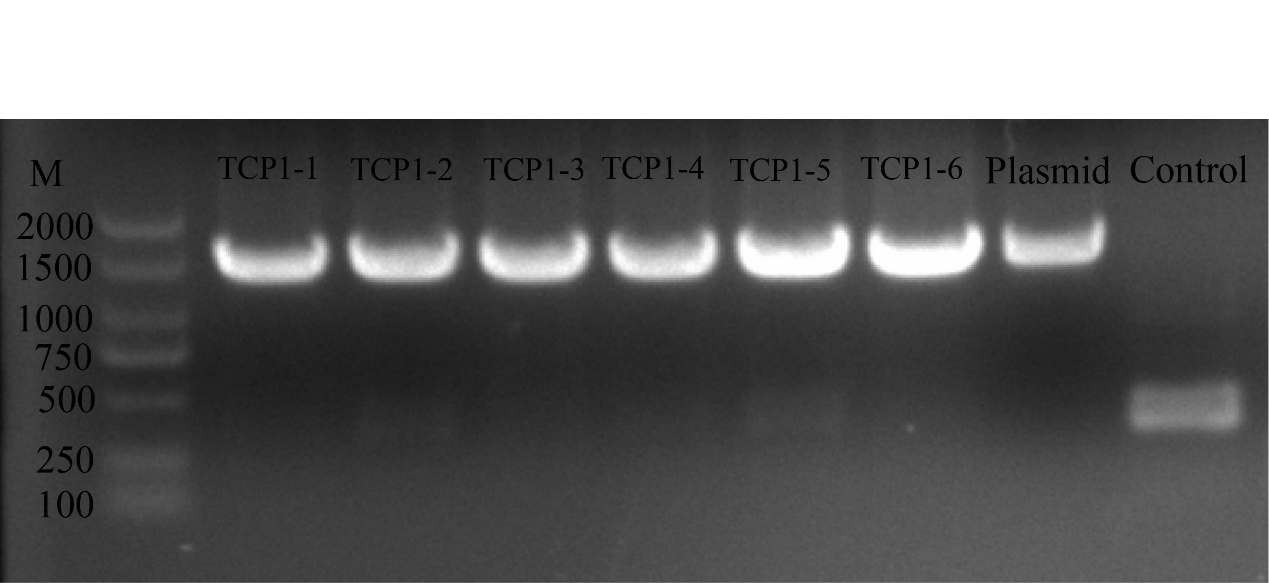


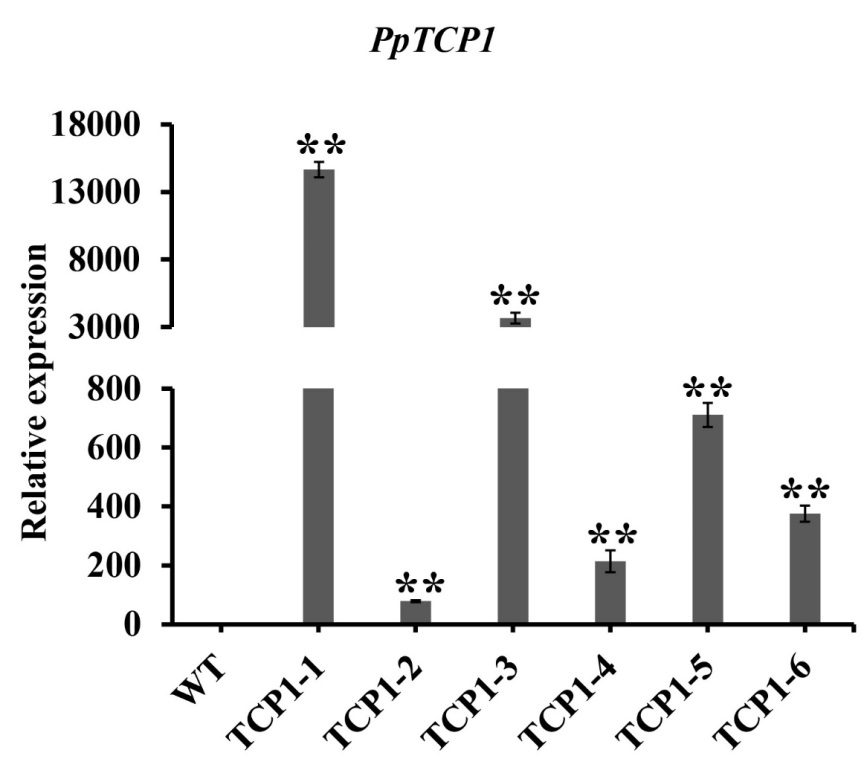


**WT TCP18-1 TCP18-2 TCP18-3 TCP18-4 TCP18-5 TCP18-6**

***PpTCP18***

(**b**)

**Figure S4.** Identification of transgenic lines overexpressing *PpTCP18*. (**a**) The six transgenic lines were screened by PCR to confirm they were transformed successfully. M: Marker DL2000. (**b**) The level of *PpTCP18* transcripts in 14-day-old Arabidopsis plants (seedlings) of WT and 35S:PpTCP18 lines were assessed by qRT-PCR.
