# Supplementary material for: PpTCP18 is upregulated by lncRNA5 and controls branch number in peach (Prunus persica) through positive feedback regulation of strigolactone biosynthesis
Source: Hortic Res. 2022 Oct 7;10(1):uhac224. doi: 10.1093/hr/uhac224 (PMC9832876; doi:10.1093/hr/uhac224)
Supplement: Web_Material_uhac224 [file web_material_uhac224.zip › Fig. S5.docx]

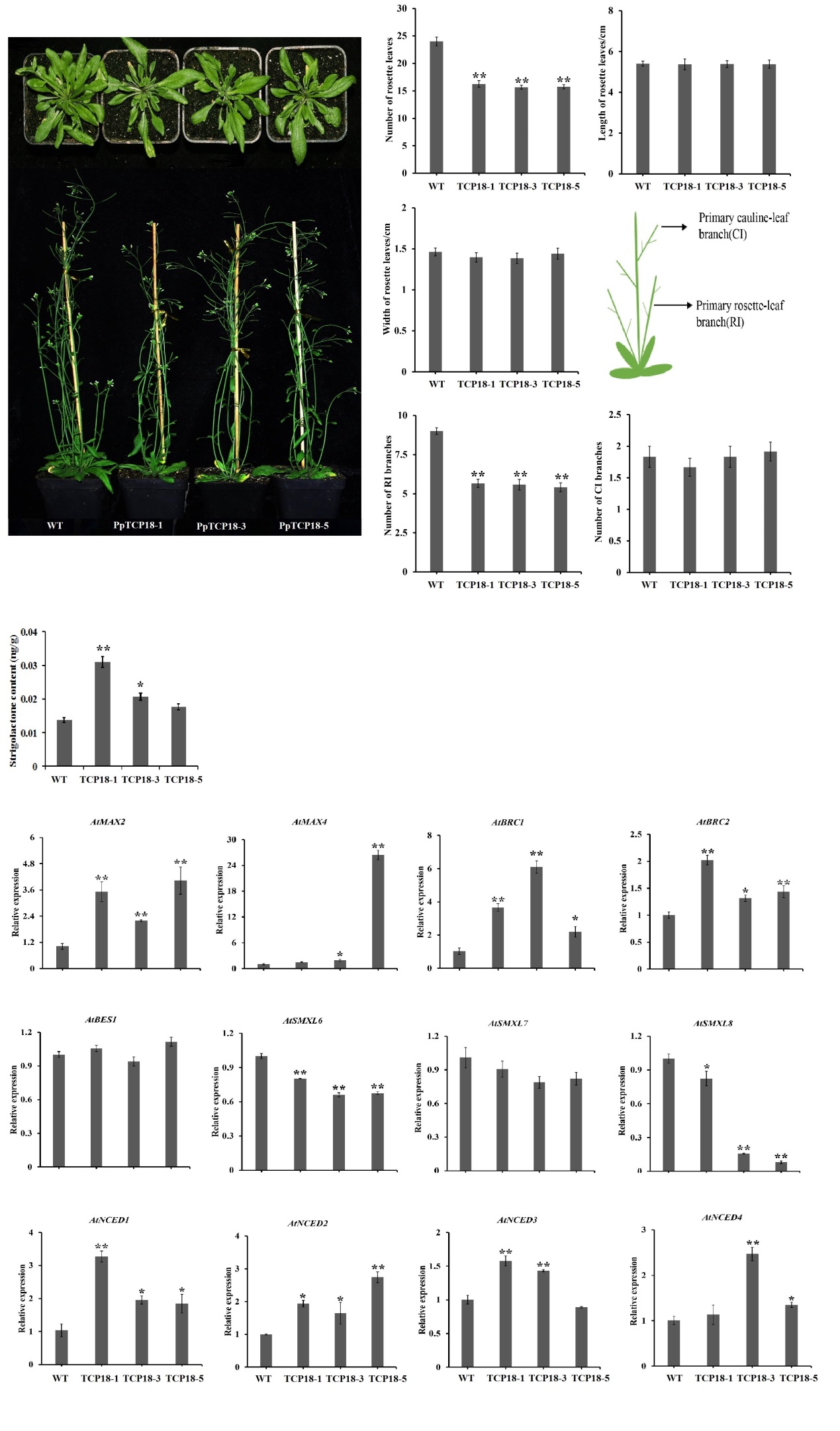


**(h)**

**(g)**

**(c)**

**(e)**

**(d)**

**(b)**

**(a)**

**(f)**

**(j)**

**(i)**

**(a)**

**(i)**

**(f)**

Fig. S5. Phenotype of PpTCP18 overexpression lines in Arabidopsis. (a) Rosette phenotype of WT, TCP18-OE1, TCP18-OE3 and TCP18-OE5. (b) Number of rosette leaves. (c/d) Length and width of rosette leaves. (**e**) Arabidopsis branching structure. (**f**) Branching phenotype of WT, TCP18-OE1, TCP18-OE3 and TCP18-OE5. (**g/h**) Number of primary cauline leaf branches (CI) and primary rosette leaf branches (RI). (**i**) SL content in WT and transgenic lines. (**j**) qRT-PCR analysis of SL-related genes and ABA synthesis genes in TCP18-OE1, TCP18-OE3, TCP18-OE5 and WT lines. Values represent the mean of three biological replicates (n = 3). Values were means ± SD of three biological replicates, ** represent significance at *P* <0.01, compared to WT or control based on *t*-test.
